# SOCIAL PARTICIPATION AND DEPRESSIVE SYMPTOMS ON THE RELATIONSHIP BETWEEN COGNITIVE FRAILTY AND DISABILITY

**DOI:** 10.1093/geroni/igad104.3733

**Published:** 2023-12-21

**Authors:** Hongting Ning, Hui Feng, Junxin LI, Mengchi Li

**Affiliations:** Central South University, Changsha, Hunan, China (People’s Republic); Central South University, Changsha, Hunan, China (People’s Republic); Johns Hopkins University, Baltimore, Maryland, United States; Johns Hopkins, Baltimore, Maryland, United States

## Abstract

To explore the multiple mediating effects of social participation and depressive symptoms on the relationship between cognitive frailty and functional disability. A total of 6122 participants from four waves of the China Health and Retirement Longitudinal Study (CHARLS) were included. Functional status was assessed by activities of daily living (ADL) and instrumental ADL (IADL). Analyses were restricted to middle-aged and older adults free of functional disability at baseline. Competing-risks regression and multiple mediation effects analysis adjusted for covariates were conducted. Cognitive frailty (hazard ratio [HR] 1.27, 95% confidence interval [CI] 1.09, 1.48), social participation (HR 0.87, 95%CI 0.79, 0.97), and depressive symptoms (HR 1.75, 95%CI 1.56, 1.95) were associated with the incidence of ADL disability. These factors were also found to predict the occurrence of IADL disability. Cognitive frailty was found to be associated with ADL disability, not only through the pathways of social participation (β = 0.009, 95% CI 0.001, 0.018) and depressive symptoms (β = 0.094, 95% CI 0.062, 0.131) separately, but also through social participation and depressive symptoms sequentially (β = 0.002, 95% CI 0.001, 0.003). Similar findings were also observed for the outcome of IADL disability. Cognitive frailty has been associated with functional disability, wherein social participation and depressive symptoms partially mediate the association. To prevent functional disability, it’s suggested to implement integrated and comprehensive intervention measures, including preventing and managing cognitive frailty, promoting social participation, and improving depressive symptoms.

